# Shall Medical Officers of the Army Engage in Private Practice?

**Published:** 1884-09

**Authors:** 


SHALL MEDICAL OFFICERS OF THE ARMY ENGAGE IN PRIVATE PRACTICE?

Again the hue and cry against officers in the army engaging in private practice is heard. *The Medical Record*, of 19th July, publishes a letter, by Dr. John G. Stanton, of New London, Conn., in which a vehement protest against this time-honored privilege is made. *Caduceus*, in *The Record*, of 19th August, ably defends the existing custom. We call attention to a vigorous letter, by a distinguished and experienced army surgeon, which appears in our columns.

The subject is worthy of consideration, since the hostility of the members of the profession in Washington to gentlemen of the Army, Navy and Marine Hospital Service, practicing in that community, is well known, and has instigated more than one abortive effort at legislative interference with the privileges of the class. Such enmity is an opprobrium to the profession. The examinations for admission into the medical corps of the army and navy are searching, thorough, rigorous, and *strictly impartial.* The Marine Hospital Service claims identity of conditions in these respects. The services are accordingly constituted of truly representative men. This fact is well recognized by the general profession, and a commission in any one of the services is properly regarded as the highest qualifying document attainable by a medical man in America. The same fact has met with abundant recognition among the laity. It is generally conceded that the Engineer and Medical Corps are the best manned organized divisions of the army. The position of the Navy Medical Corps, at the head of the Staff, has been equally well sustained. Very recently, and as the resultant of strenuous efforts, the Marine Hospital Service has achieved a similarly lofty eminence.

As scientific investigators, medical and surgical practitioners, gallant officers, and well-bred gentlemen, the medical officers of the Army, Navy and Marine Hospital Service reflect credit upon the American medical profession and have well earned the universal esteem in which they are held. The very least the profession can do is to leave the medical officers of the United States government in full possession of the limited privileges which they at present enjoy.

In the words of Dr. Heiskell:

"It may not be out of place incidentally to state that to prohibit a medical officer (when his public duties will permit) from extending relief to those of his fellow-citizens who may apply for his services—having confidence in his professional attainments—would be as ungracious to them as it would be devoid of the common dictates of humanity, and might afford as just and perhaps a better cause of complaint on the part of the neighboring community than the one alleged by yourselves, which relates exclusively to private interests."

